# Optimization of Extraction Condition of Bee Pollen Using Response Surface Methodology: Correlation between Anti-Melanogenesis, Antioxidant Activity, and Phenolic Content

**DOI:** 10.3390/molecules201119656

**Published:** 2015-11-02

**Authors:** Seon Beom Kim, Yang Hee Jo, Qing Liu, Jong Hoon Ahn, In Pyo Hong, Sang Mi Han, Bang Yeon Hwang, Mi Kyeong Lee

**Affiliations:** 1 College of Pharmacy, Chungbuk National University, Cheongju, Chungbuk 28644, Korea; suntiger85@hanmail.net (S.B.K.); qow0125@naver.com (Y.H.J.); liuqing7115@hotmail.com (Q.L.); zzonggoo07@naver.com (J.H.A.); byhwang@chungbuk.ac.kr (B.Y.H.); 2 National Academy of Agricultural Science, Rural Development Administration, Jeonju, Chonbuk 54875, Korea; iphong20@korea.kr (I.P.H.); sangmih@korea.kr (S.M.H.)

**Keywords:** bee pollen, oxidative stress, melanogenesis, optimization, phenolic content, response surface methodology

## Abstract

Bee pollen is flower pollen with nectar and salivary substances of bees and rich in essential components. Bee pollen showed antioxidant and tyrosinase inhibitory activity in our assay system. To maximize the antioxidant and tyrosinase inhibitory activity of bee pollen, extraction conditions, such as extraction solvent, extraction time, and extraction temperature, were optimized using response surface methodology. Regression analysis showed a good fit of this model and yielded the second-order polynomial regression for tyrosinase inhibition and antioxidant activity. Among the extraction variables, extraction solvent greatly affected the activity. The optimal condition was determined as EtOAc concentration in MeOH, 69.6%; temperature, 10.0 °C; and extraction time, 24.2 h, and the tyrosinase inhibitory and antioxidant activity under optimal condition were found to be 57.9% and 49.3%, respectively. Further analysis showed the close correlation between activities and phenolic content, which suggested phenolic compounds are active constituents of bee pollen for tyrosinase inhibition and antioxidant activity. Taken together, these results provide useful information about bee pollen as cosmetic therapeutics to reduce oxidative stress and hyperpigmentation.

## 1. Introduction

Skin interfaces with the environment and is the primary target of environmental stresses. Exposure to repeated UV irradiation and pollution produces oxidative stress to skin which, consequently, causes skin damage and the aging process. Antioxidant defense system in our body such as superoxide dismutase, catalase, and glutathione reduces reactive oxygen stress (ROS) and protects our skin from oxidative stress [1,2,3,4]. Melanin is a dark macromolecular pigment produced in melanocytes by melanogenesis. It also plays an important role in protecting skin from UV radiation and toxic chemicals, however, excessive production and accumulation of melanin produces ROS, which aggravates skin damage. Moreover, excessive and uneven accumulation of melanin in specific parts induces pigmentation problems [5,6]. Therefore, natural products which reduce oxidative stress and inhibit melanin synthesis have become important constituents in cosmetic products [7,8,9,10].

Bee pollen is flower pollen collected by bees, which results in the agglutination of pollen with nectar and salivary substances of bees. It is rich in amino acids, proteins, hormones, minerals, and vitamins, which contribute to the beneficial effects on the immune defense system, as well as anti-aging, antioxidant, antimicrobial, anti-inflammatory, and chemopreventive activity [11,12,13]. Although the chemical composition of bee pollen is varied depending on plant sources, it contains high amounts of phenolic constituents, such as flavonoids, anthocyanins, and tannins, which exhibit biological activity [14,15,16,17]. Due to diverse beneficial effects, bee pollen is widely consumed and commercially available as dietary supplements all over the world.

**Figure 1:**
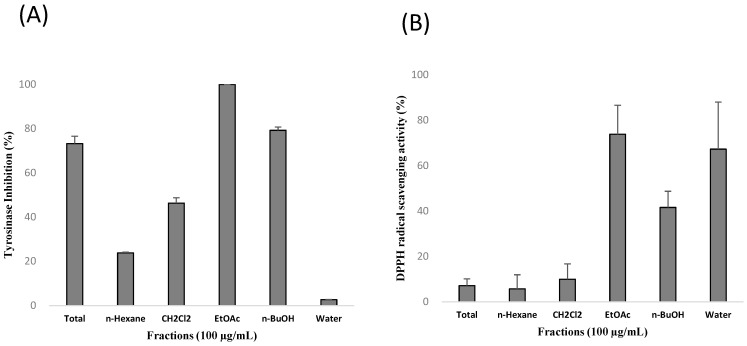
Effect of total extract and each fraction of bee pollen on (**A**) tyrosinase inhibition and (**B**) radical-scavenging activity.

Bee pollen is well known for antioxidant effect [15,16,17]. In our present study, bee pollen also inhibited tyrosinase activity, a key enzyme in melanin synthesis (Figure 1). Therefore, bee pollen is suggested as a promising therapeutic candidate for skin aging by melanogenesis inhibitory and antioxidant effect. For development of cosmetic products, extraction of bee pollen is required. During extraction, many extraction factors such as extraction solvent, extraction time, extraction temperature, and solid-liquid ratios affect the composition of extract, as well as its biological activity [18,19,20]. Therefore, optimization of extraction condition is required for maximum efficacy. Response surface methodology (RSM), a mathematical and statistical tool, takes several factors into account simultaneously using rationally-designed experiments. It can derive the optimal condition effectively, especially in the case of several variables [21,22,23]. Therefore, RSM is widely used for optimization of extraction conditions in many fields.

In the present study, anti-melanogenesis and antioxidant activity of bee pollen were investigated using tyrosinase and a DPPH radical-scavenging assay system, respectively. For optimization, response surface methodology with three-level-three-factor Box-Behnken design (BBD) was employed to evaluate the effect of multiple factors of extraction condition on tyrosinase inhibition and antioxidant activity. The correlation between activities and phenolic content was also analyzed.

## 2. Results and Discussion

### 2.1. Anti-Melanogenesis and Antioxidant Activity of Bee Pollen Fractions

The MeOH extract of bee pollen was fractionated into *n*-hexane, CH_2_Cl_2_, EtOAc, and *n*-BuOH fraction, and the anti-melanogenesis and antioxidant activity were evaluated. Among fractions, EtOAc-soluble fractions showed the most potent activity for both tyrosinase inhibition and radical scavenging activity. The *n*-BuOH-soluble fraction also inhibited tyrosinase activity, and *n*-BuOH and water-soluble fractions showed radical-scavenging activity. *n*-Hexane and CH_2_Cl_2_ fractions, however, showed weak activity in our assay system (Figure 1).

### 2.2. Optimization of Extraction Condition

Since the anti-melanogenesis and antioxidant activity of bee pollen were quite different between each solvent fraction, we attempted to optimize the extraction conditions for maximum tyrosinase inhibition and antioxidant activity.

#### 2.2.1. Extraction Method Development

The optimized extraction condition of bee pollen for tyrosinase inhibition and antioxidant activity was investigated using response surface methodology employing Box-Behnken design (BBD) with three-level-three-factor in the present study.

Extraction solvent, extraction temperature, and extraction time were chosen for extraction variables and the range of each variable was determined on the preliminary study. Since the EtOAc-soluble fraction and the *n*-BuOH soluble fraction showed the most potent activity in both tyrosinase inhibition and radical-scavenging activity (Figure 1), the extraction solvent was chosen by the combination with EtOAc and MeOH. The range of extraction temperature and extraction time was determined on the basis of preliminary single factor experiment.

Taken together, extraction variables for response surface methodology were selected as extraction solvent (*X*_1_, EtOAc concentration in MeOH, 50%, 75% and 100%), extraction temperature (*X*_2_, 10, 30 and 50 °C), and extraction time (*X*_3_, 19, 31, and 43 h). The variables were coded at three levels (−1, 0, and 1) and the complete design consisted of 15 experimental points, including three replications of the center points (all variables were coded as zero), as shown in Table 1. Tyrosinase inhibition and antioxidant activity were greatly affected depending on extraction conditions. Tyrosinase activity (%) was ranged from 18.4% to 57.9% and antioxidant activity (%) was ranged from 12.7% to 50.1% depending on 15 experimental points (Table 1), which showed the importance for optimization of extraction condition.

**Table 1:** A Box-Behnken design for independent variables and their responses.

Run	Actual Variables			Observed Values		
	EtOAc in MeOH (%)	Extraction Temperature (°C)	Extraction Time (h)	Tyrosinase Inhibition ^a^ (%)	Antioxidant Activity ^b^ (%)	Total Phenolic (μg GAE/mg Extract)
1	100	30	43	29.8	14.4	8.7
2	50	30	43	42.2	31.1	13.4
3	75	50	19	49.1	37.9	16.8
4	75	30	31	53.9	50.1	20.4
5	100	50	31	29.7	12.7	7.5
6	75	30	31	49.1	46.5	18.4
7	75	30	31	55.9	46.7	19.3
8	75	50	43	50.6	38.6	17.7
9	100	30	19	28.3	12.7	8.0
10	50	50	31	32.3	25.4	13.6
11	50	10	31	45.7	32.8	14.1
12	50	30	19	40.8	32.9	13.8
13	75	10	43	57.9	42.0	18.4
14	75	10	19	55.3	48.5	20.4
15	100	10	31	18.4	13.0	7.4

^a^ Tyrosinase inhibition (%) was measured at 100 μg/mL. ^b^ Antioxidant activity (%) was measured at 300 μg/mL.

#### 2.2.2. Fitting the Models

**Figure 2:**
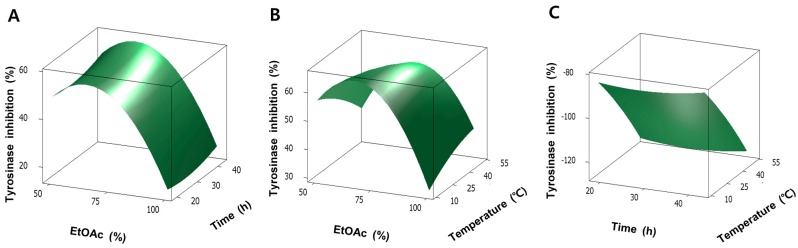

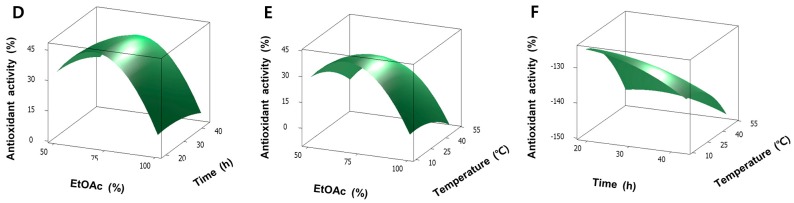
Response surface plots and contour plots show the effect of extraction variables on tyrosinase inhibition (**A**–**C**) and antioxidant activity (**D**–**F**). Three variables are EtOAc concentration (*X*_1_), extraction time (*X*_2_), and extraction temperature (*X*_3_).

Second-order polynomial regression equations were established by RSM for the evaluation of the relationship between variables and responses. Greater coefficients with smaller *p*-value (*p* < 0.05) indicated the considerable effect of these coefficients on respective responses (Table 2). The values of multiple determination (*R*^2^) were 0.979 and 0.993 for tyrosinase inhibition and antioxidant activity, respectively, which demonstrated effectiveness of this model (Table 3). Insignificant *p*-value of lack of fit, 0.697 and 0.556 for tyrosinase inhibition and antioxidant activity, respectively, also indicated the adaptability of this model to experimental data (Table 3). The relationship between every two variables in tyrosinase inhibition and antioxidant activity was shown in three-dimensional response surface plots based on regression equations (Figure 2). Collectively, this model is adequately fitted to experimental data and suitable for optimization. 

**Table 2:** Regression coefficients and their significances in the second-order polynomial regression equation.

	Coefficient	Standard Error	*t* Value	*p* Value
(Tyrosinase Inhibition)				
Intercept	52.96	1.707	31.032	<0.001
*X*_1_	−6.868	1.045	−6.571	0.001
*X*_2_	0.883	1.045	0.844	0.437
*X*_3_	−1.943	1.045	−1.859	0.122
$X_{1}^{2}$	−19.69	1.538	−12.8	<0.001
$X_{2}^{2}$	2.015	1.538	1.31	0.247
$X_{3}^{2}$	−1.755	1.538	−1.141	0.306
*X*_1_*X*_2_	0.04	1.478	0.027	0.979
*X*_1_*X*_3_	0.16	1.478	4.168	0.009
*X*_2_*X*_3_	−0.315	1.478	−0.213	0.84
(Antioxidant Activity)				
Intercept	47.473	1.15	41.534	<0.001
*X*_1_	−8.663	0.704	−12.306	<0.001
*X*_2_	-0.75	0.704	−1.065	0.335
*X*_3_	−2.723	0.704	−3.868	0.012
$X_{1}^{2}$	−22.884	1.036	−22.086	<0.001
$X_{2}^{2}$	−2.089	1.036	−2.016	0.1
$X_{3}^{2}$	−3.889	1.036	−3.754	0.013
*X*_1_*X*_2_	0.9	0.996	0.904	0.407
*X*_1_*X*_3_	1.755	0.996	1.763	0.138
*X*_2_*X*_3_	1.8	0.996	1.808	0.13

**Table 3:** ANOVA for response surface regression equation.

	Sum of Square	Degree of Freedom	Mean Square	*F* Value	*p* Value
(Tyrosinase inhibition)					
Regression	2044.58	9	227.175	26	0.001
Linear	413.72	3	137.906	15.78	0.006
Square	1478.67	3	492.891	56.41	<0.000
Interaction	152.19	3	50.729	5.81	0.044
Residual error	43.69	5	8.737		
Lack-of-fit	19.72	3	6.573	0.55	0.697
Pure error	23.97	2	11.985		
Total	2088.26	14			
*R*^2^ = 0.979, adjusted *R*^2^ = 0.941					
(Antioxidant activity)					
Regression	2643.18	9	293.687	74.09	<0.001
Linear	664.11	3	221.369	55.84	<0.001
Square	1950.56	3	650.185	164.02	<0.001
Interaction	28.52	3	9.507	2.4	0.184
Residual error	19.82	5	3.964		
Lack-of-fit	11.54	3	3.848	0.93	0.556
Pure error	8.28	2	4.138		
Total	2663	14			
*R*^2^ = 0.993, adjusted *R*^2^ = 0.979					

#### 2.2.3. Effect of Extraction Variables on Tyrosinase Inhibition and Antioxidant Activity

Multiple regression analysis on the experiment data yielded the second-order polynomial regression equation for coded values as follows:

(1) $$\text{Tyrosinase inhibition}(\%)=52.96-6.87X_{1}+0.88X_{2}-1.94X_{3}-19.69X_{1}^{2}+2.02X_{2}^{2}-1.76X_{3}^{2}+0.04X_{1}X_{2}-0.16X_{1}X_{3}-0.32X_{2}X_{3}$$

(2) $$\text{Antioxidant activity}(\%)=47.47-8.66X_{1}-0.75X_{2}-2.72X_{3}-22.88X_{1}^{2}-2.09X_{2}^{2}-3.89X_{3}^{2}+0.90X_{1}X_{2}+1.76X_{1}X_{3}+1.80X_{2}X_{3}$$

Among extraction variables, the linear (*X*_1_) and quadratic term ($X_{1}^{2}$) of EtOAc concentration showed the most significant effect on both tyrosinase inhibition and antioxidant activity with *p*-value of <0.001 (Table 2). The interaction term of EtOAc concentration and extraction temperature (*X*_1_*X*_3_) also showed significant effect on tyrosinase inhibition (*p* value of 0.009). Antioxidant activity was significantly affected by the linear (*X*_3_) and quadratic term of extraction temperature ($X_{3}^{2}$) with *p* values of 0.012 and 0.013, respectively. Other variables including extraction time, however, did not show any significant effect in our present study.

The *F*-values of 26.00 and 74.09, together with *p*-values of 0.001 and <0.001 for the tyrosinase inhibition and antioxidant activity, respectively, indicated that the model adequately fitted the experimental data. In addition, insignificant *p*-value for lack-of-fit as 0.697 and 0.556 for the tyrosinase inhibition and antioxidant activity, respectively, also supported the fitness of the model (Table 3).

Three-dimensional response surface plots showed similar patterns for tyrosinase inhibition and antioxidant activity. Consistent with multiple regression analysis, extraction solvent showed the dramatic effect on tyrosinase inhibition and antioxidant activity. On fixed temperature at 30 °C, EtOAc concentration exert quadratic effect on the tyrosinase inhibition (Figure 2A) and antioxidant activity (Figure 2D). Tyrosinase inhibition and antioxidant activity increased with increasing EtOAc concentration but decreased with continuing increase of EtOAc concentration. A similar quadratic pattern of EtOAc concentration was observed at fixed time (Figure 2B,E). However, compared to extraction solvent, tyrosinase inhibition and antioxidant activity showed slight changes as extraction time and temperature increased (Figure 2).

### 2.3. Optimization of Extraction Parameters and Verification

Based on our results, an optimization for extraction condition for both responses was determined by RSM and verified by experiment. Optimal extraction condition of bee pollen for maximum tyrosinase inhibition and antioxidant was determined as EtOAc concentration in MeOH, 69.6%, temperature of 10.0 °C, and extraction time, 24.2 h, which predicted 55.0% tyrosinase inhibition and 48.6% antioxidant activity (Table 4). Bee pollen extract prepared under optimized condition showed 54.9% tyrosinase inhibition and 44.1% antioxidant activity, which was well-matched with predicted values.

**Table 4:** Predicted and observed values of tyrosinase inhibition and antioxidant activity under optimized condition.

Extraction Condition			Tyrosinase Inhibition ^a^		Antioxidant Activity ^b^	
EtOAc in MeOH (%)	Extraction Temperature (°C)	Extraction Time (h)	Predicted	Observed	Predicted	Observed
69.6	10.0	24.2	55.0	57.9	48.6	49.3

^a^ Tyrosinase inhibition (%) was measured at 100 μg/mL. ^b^ Antioxidant activity (%) was measured at 300 μg/mL.

### 2.4. Correlation between Activity and Phenolic Content

**Figure 3:**
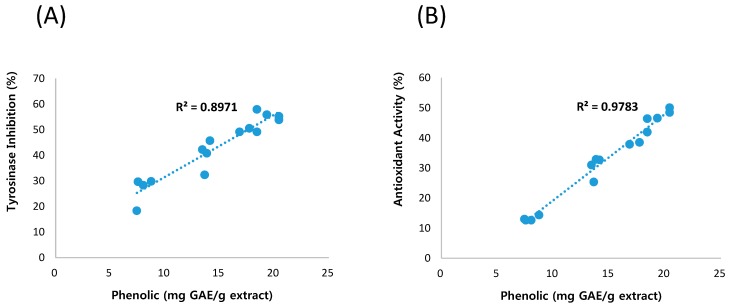
Correlation between tyrosinase inhibition and phenolic content (**A**) and between antioxidant activity and phenolic content (**B**).

Bee pollen contains various phenolic constituents including flavonoids, phenolic acid, anthocyanins, and tannin [15,16,17]. Phenolic constituents are known to exert diverse biological activities, including antioxidant and anti-melanogenesis activity [10,24,25]. Therefore, correlations between each response and phenolic content were investigated. As shown in Figure 3, tyrosinase inhibition and antioxidant activity were highly proportional to phenolic content with *R*^2^ values of 0.897 and 0.973, respectively (Figure 3). Based on these results, we suggested that anti-melanogenesis and antioxidant activity of bee pollen were mainly achieved by phenolic constituents. In addition, phenolic content of bee pollen can be used for the quality control of products.

### 2.5. Discussion

Bee pollen is flower pollen with nectar and salivary substances of bees and considered as perfect foods due to its nutritional value. It is rich in essential components, including amino acids, proteins, hormones, minerals, and vitamins, and exerts diverse beneficial effect on humans [11,12,13,26,27,28]. 

Oxidative stress results from an imbalance between the production of reactive oxygen species and antioxidant defenses and is considered as a major contributor to age-related symptoms and pathogenesis of many diseases. Skin is vulnerable to oxidative stress, and exposure to repeated oxidative stress contributes to skin aging [1,2,3,4]. Although melanin protects skin from oxidative stress, accumulation of excessive melanin in specific parts causes pigmentation problems and important issues in cosmetic field [5,6,29]. Therefore, inhibition of oxidative stress and excessive melanin accumulation is one of the important strategies in cosmetic industry. Bee pollen is well known for its antioxidant effect [14,15,16]. In addition, bee pollen inhibited tyrosinase activity in our present study. Therefore, bee pollen is expected to be beneficial to skin problems in combinatorial action and might be a good resource in cosmetic products.

For the development of bee pollen as cosmetics, the extraction condition is important for maximum efficacy. Our present study clearly showed the crucial effect of extraction conditions on tyrosinase inhibitory activity and antioxidant activity (Table 1). Especially, tyrosinase inhibitory activity and antioxidant activity of bee pollen was greatly affected by the extraction solvent (Figure 2). Optimal extraction condition was derived from RSM and extract prepared from this condition showed similarity with predicted values (Table 4).

Phenolic compounds including flavonoids, phenolic acids are reported as major compounds of bee pollen and contributed to its diverse biological activity [14,15,16,17]. Consistent with previous studies, bee pollen extract is rich in phenolic compounds. The amounts of phenolic compounds were, however, different depending on extraction conditions ranging from 7.4 to 20.4 μg/mg extract (Table 1). Further analysis between tyrosinase inhibition and phenolic content showed close correlation with *R*^2^ value of 0.897 (Figure 3A). Tyrosinase inhibitory activity was increased with increasing phenolic contents. Antioxidant activity was also proportional to the amount of phenolic compounds (Figure 3B). These results suggested phenolic compounds as active constituents of bee pollen for tyrosinase inhibition and antioxidant activity. Therefore, the content of phenolic compounds can be used as a quality control for the development of cosmetics.

## 3. Experimental Section

### 3.1. General Information

#### 3.1.1. Bee Pollen 

Bee pollen was directly collected from a pollen trap in Paju, Gyeonggi Province, and was provided by the Rural Development Administration of Korea (Chonbuk, Korea). The freshly-harvested bee pollen was dried at 40 °C and then stored in a freezer until use. The dominant plants surrounding the farm were acorn trees (*Quercus acutissima*, Fagaceae). Identification was conducted by Dr. In Pyo Hong (Rural Development Administration of Korea) using color and scanning electron microscope (SEM) analysis, supporting acorn bee pollen as the principal pollen.

#### 3.1.2. Preparation of Extract and Fractions 

Bee pollen was extracted twice with 80% MeOH, which yielded the total extract. The total MeOH extract was then suspended in H_2_O and partitioned successively with *n*-hexane, CH_2_Cl_2_, EtOAc, and *n*-BuOH and yielded *n*-hexane, CH_2_Cl_2_, EtOAc, *n*-BuOH, and water-soluble fractions.

### 3.2. Response Surface Methodology

A Box-Behnken design (BBD) with three variables and three levels was used to optimize the extraction conditions of bee pollen. Target responses were selected for tyrosinase inhibition and antioxidant activity. For independent extraction variables, extraction solvent, EtOAc in MeOH (*X*_1_), extraction time (*X*_2_), and extraction temperature (*X*_3_) were chosen in this study and the ranges of these variables were determined on the basis of a preliminary single factor experiment. As shown in the complete design consisted of 15 experimental points, including three replications of the center points (all variables were coded as zero).

Regression analysis was performed according to the experimental data. The mathematical model can be explained by the following equation:

(3) $$Y=\beta_{0}+\overset{3}{\underset{i=1}{\sum }}\beta_{i}X_{i}+\;\overset{3}{\underset{i=1}{\sum }}\beta_{ii}X_{i}^{2}+\overset{3}{\underset{1\leq i\leq j}{\sum }}\beta_{ij}X_{i}X_{j}$$

where *Y* is the response, β_0_ is the constant coefficient, β*_i_* are the linear coefficients, β*_ii_* are the quadratic coefficients, and β*_ij_* are the interaction coefficients. The statistical significance of the coefficients in the regression equation was checked by analysis of variance (ANOVA). The fitness of the polynomial model equation to the responses was evaluated with the coefficients of *R*^2^ and the lack of fit was evaluated using *F*-test.

### 3.3. Evaluation of Anti-Melanogenesis and Antioxidant Activity

#### 3.3.1. Preparation of Samples

Test samples were prepared as indicated and the solvent was removed *in vacuo*. For the assessment of biological activity, the lyophilized samples were first dissolved in dimethyl sulfoxide (DMSO) and then diluted in PBS. The final concentration of DMSO was adjusted less than 0.1%. The effect of DMSO was tested using a vehicle-treated control and did not affect biological activity in our assay system.

#### 3.3.2. Assessment of Tyrosinase Activity 

Tyrosinase inhibitory assays were performed using an enzyme solution, which was prepared by the reconstitution of mushroom tyrosinase (Sigma, St. Louis, MO, USA) in 100 U/mL phosphate buffer (pH 6.5). Test samples were mixed with 50 μL enzyme buffer, and incubated for 5 min at 37 °C. Then, 50 μL tyrosine solution, which was diluted with phosphate buffer to 1 mM, was added and the enzyme reaction was allowed to proceed for 20 min at 37 °C. After incubation, the amount of dopachrome formed in the reaction mixture was determined by measuring the absorbance at 490 nm in an ELISA reader.

#### 3.3.3. Measurement of Antioxidant Activity

The antioxidant activity was evaluated by measuring the free radical scavenging activity using 2,2-diphenyl-1-picrylhydrazyl (DPPH). Briefly, extracts prepared from different extraction conditions were mixed with freshly prepared DPPH solution. After shaking, the reaction mixtures were left to stand for 30 min at room temperature in the dark. The radical-scavenging activity was determined by measuring the absorbance at 517 nm. The relative radical scavenging activity (%) was calculated as [1 − absorbance of solution with sample and DPPH/absorbance of solution with DPPH] × 100.

### 3.4. Measurement of Total Phenolic Content

The total phenolic content was measured with a Folin-Ciocalteau assay. Folin-Ciocalteau’s phenol reagent was added to the 96-well plate containing the test samples. After 5 min of incubation with gentle shaking, 7% Na_2_CO_3_ was added to the reaction mixture. The reaction mixture was left in the dark for 90 min at room temperature. The absorbance was measured at 630 nm with a microplate reader. The total phenolic content was expressed as gallic acid equivalent (GAE) using gallic acid (0.02 mg/mL − 1.2 mg/mL) as a standard.

## 4. Conclusions

Bee pollen exerted tyrosinase inhibition and antioxidant activity. For maximum efficacy, optimal extraction conditions were derived using response surface methodology, with EtOAc concentration in MeOH, 69.6%, temperature, 10.0 °C, and extraction time, 24.2 h, which predicted 55.0% tyrosinase inhibition and 48.6% antioxidant activity. Bee pollen extract prepared from optimized condition showed 57.9% tyrosinase inhibition and 49.3% antioxidant activity, which was well-matched with predicted values. Thus, this model can be used to optimize extraction process of bee pollen. Tyrosinase inhibition and antioxidant activity of bee pollen is closely correlated with the amount of phenolic content, which suggested phenolic compounds are active constituents. Taken together, these results provide useful information about bee pollen as cosmetic therapeutics to reduce oxidative stress and hyperpigmentation.
